# The chameleon effect in customer relationship management: Experiments on the spillover effects of mimicry in natural settings of a chain hotel and a chain grocery shop

**DOI:** 10.3389/fpsyg.2023.1016125

**Published:** 2023-03-14

**Authors:** Wojciech Kulesza, Dariusz Dolinski, Paweł Muniak, Joanna Borkowska, Polina Bibikova, Tomasz Grzyb

**Affiliations:** ^1^Faculty of Psychology in Warsaw, Centre for Research on Social Relations, SWPS University of Social Sciences and Humanities, Warsaw, Masovian, Poland; ^2^Faculty of Psychology in Wroclaw, SWPS University of Social Sciences and Humanities, Warsaw, Masovian, Poland; ^3^Faculty of Psychology in Warsaw, SWPS University of Social Sciences and Humanities, Warsaw, Masovian, Poland

**Keywords:** chameleon effect, verbal mimicry, spillover effect, service quality measurement, hospitality management

## Abstract

Numerous experiments have proven that mimicry is highly beneficial (mainly to the mimicker but also to the mimickee). Some studies have shown initial data suggesting the potential of applying this knowledge to business settings. In the present paper we unpack this issue in two ways. First, by presenting potential benefits stemming from mimicry for the mimicking dyad, and second for the business environment represented by the mimicker. Two consecutive studies: a Pretest and a Main Experiment run in natural settings showed great potential in improving the assessments of quality of service provided by verbally mimicking (or not). The results of both studies showed that mimicry offers benefits for the mimicker (increased employee kindness and employee evaluation), and also spillover to the organization/company represented by the mimicking employee (increased opinion of and willingness to return to the shop/hotel). Future research directions and limitations are discussed.

## 1. Introduction

It seems that imitation and mimicry are present between almost all creatures on Earth. Insects, for example, employ mimicry to avoid being attacked by predators (Hossie and Sherratt, 2013). Additionally, more developed animals, like wolves and cats, use mimicry to survive and thrive (Range and Virányi, 2014). It has been shown, for example, that small cats imitate their mothers when choosing a food to eat: they eat exactly what their mothers eat (e.g., Wyrwicka and Long, 1980). In the same vein, human children imitate their parents while learning social behaviors (like aggression) and social roles (Bandura, 1962; Bandura et al., 1963). It has also been shown that children imitate each other, for example, by mimicking crying (Simner, 1971) and facial expressions (Termine and Izard, 1988). Mimicry was mainly developed on the grounds of clinical psychology. For example, Charny (1966) found that patients and therapists tended to imitate each other, which led to a rapport (Scheflen, 1964; Lafrance and Broadbent, 1976; Maurer and Tindall, 1983).

It was also shown that people tend to imitate each other in more general social situations and imitation leads to benefits for the mimicker, namely increased liking granted by the mimickee (Chartrand and Bargh, 1999). This effect was coined “the chameleon effect.” Further studies on the effects stemming from mimicry have shown that mimicry is highly beneficial. For example, it has been shown that mimicry impacts trust (Guéguen et al., 2013; Shaw et al., 2015; Novotny et al., 2021), perceptions of the physical attractiveness of the mimicker (Guéguen, 2009) as well as the attractiveness of the out-group represented by the mimicker (Zglinicka and Kulesza, 2014), feeling that the interaction provides a better connection and is smoother (Chartrand and Bargh, 1999; Zhou and Fischer, 2018; Yokotani et al., 2020), the tendency to provide help to the mimicker (Van Baaren et al., 2004; Kulesza et al., 2014a), and the perception that the world is as a fairer place (Stel et al., 2013). Mimicry also changes the perception of victims of violent crime, decreasing blame assigned to the victim (Stel et al., 2012), as well as changing political views to more prosocial (Stel and Harinck, 2011). The present paper answers two questions: First, are these positive evaluations restricted to the mimicking dyad, and second can mimicry impact the perception of the social business environment represented by the mimicker?

### 1.1. Research on mimicry run in laboratories/simulations with a business potential

Mimicry is beneficial not only from a social point of view (mimicry as a social glue hypothesis; Lakin et al., 2003; Dijksterhuis, 2005) but can also be applied in corporate settings: it boosts trust in negotiations as well as influences buying tendencies and the evaluation of products. Below we elaborate on these issues in detail.

#### 1.1.1. Trust and mimicry research

Mimicry research clearly shows that this behavior leads to boosted trust. This was shown in an experiment where two members of a dyad were assigned to either the role of a person applying for a job or a recruiter (Swaab et al., 2011). The recruiter mimicked (or not) the applicant’s words at the beginning or towards the end of the interaction. The dyad benefitted more when mimicry occurred early. In the second experiment, these effects were replicated in a different cultural setting, pointing to the universality of the described relationships. The same scenario was used by Maddux et al. (2008) by showing that trust built on performed mimicry reaches a much deeper impersonal level than previously described. In the first experiment, a dyad of participants worked in one of three ways. In the first two, one person always imitated the non-verbal behavior of the other. In the last condition neither of the dyad performed mimicry. The results showed that mimicry led to higher gains for mimickers and cumulatively for the pair compared to the control condition. No benefits for mimickees were noted. In the second experiment a dyad negotiated the sale of a gas station. Interestingly, an agreement was impossible to reach. The buyer could offer no more than half a million dollars whilst the seller could not sell for less than $ 553,000 making this situation intractable. There was, however, a way out from this stalemate. The situation was arranged in such a way that the seller was interested in staying in the business as an employee which might be interesting for a buyer too (since s/he was interested in buying not only the gas station but also the existing know-how). In this case the seller might downsize his/her financial expectations in order to be hired later on. The seller’s secret was, however, the motive for selling the business: professional burnout. S/he needed to sell as quickly as possible, to relax and recuperate, and then if possible/agreed upon during negotiations – find a new job. It was measured to what extent the seller would trust the buyer by revealing this intimate information. Results showed that an agreement was reached in most pairs (10 out of 15) when mimicry was employed by the buyer in the conversation. Thus, this experiment clearly shows that trust may reach the level of being able to share intimate information with the mimicker.

In the vein of trust and at the deeper level of interpersonal relationships it was shown that not only personal intimate information is shared as a result of performed mimicry but it seems that rapport is responsible for the all aforementioned effects. To prove this relationship, participants were instructed to either mimic each other’s words, for only one participant to mimic the other, or neither (Muir et al., 2020). Results have shown that mimicry was associated with greater gains (both joint and individual), and a perception of rapport by the mimicked partner.

#### 1.1.2. Buying tendencies, evaluations of products and mimicry research

Mimicry also creates tendencies toward buying behaviors. Participants watched commercials during which participants were asked to mimic (or not) the people visible in advertisements (Stel et al., 2011a). It was found that the mimicking participants liked the presented products more than the non-mimicking ones. In the second experiment, willingness to buy the advertised products and services was measured: business spill-over effects of mimicry, participants also declared if they would like to buy other/similar products and services not presented in the watched (and mimicked or not) commercials. The results showed mimicry created a willingness to buy only the advertised products, and did not create positive evaluations of products not represented in the commercials.

In a study by Tanner et al. (2008), marketing opinions on an isotonic drink as a result of mimicry were researched, which may be extended to the business practice context. The participants were verbally and nonverbally imitated (or not) by a marketer: a person conducting the marketing research. It turned out that opinions about isotonic drinks became more positive when the marketer employed mimicry in the discussion. In the second experiment this result was replicated. Mimicry led to more positive opinions concerning isotonic soda, even when participants were notified that the marketer has a personal interest in this research. Treating the described work as a launching pad, another team wanted to investigate if mimicry changes the initial opinion of the drink to a more positive one (if an initial assessment was negative, it would become more favorable after imitation), or strengthens the existing one (if an initial assessment was negative, it would become even less favorable after imitation; Kulesza et al., 2017). To test this, participants were provided with poor-tasting isotonic drinks and during testing as well as the interview they were mimicked (or not) by the marketer. It turned out that mimicry led to more positive judgments concerning the taste of the distasteful isotonic drink when mimicry was introduced.

#### 1.1.3. Mimicry boosts persuasiveness

It was also shown that mimicry is a powerful social mechanism because it boosts susceptibility to social influence. In the first work describing this very issue, participants had to make a personal decision (Van Swol, 2003), and in the next step were asked to discuss their choice with two other people (confederates). The first confederate was supposed to agree with the study participant’s choice, whilst the second one would disagree. Both confederates mimicked (or not) the participant’s gestures. The results showed that a person who mimicked was perceived as more persuasive. The relationship between mimicry and persuasion was tested in an experiment in which participants were asked to rate to what extent they would support the idea of building a pub on a university campus (Drury and Van Swol, 2005). Participants were asked to provide a justification for their opinion and a 10 min discussion with the confederate followed. The confederate’s task was to express an opinion different from that of the participant and to imitate (or not) her/his gestures. Whether participants changed their opinion was then measured. Again, it turned out that a relationship was found for persuasiveness: mimickers were considered to be more persuasive. Taken together, mimicry seems to be a very powerful social mechanism which is beneficial not only in social relations, but may be employed (with benefits) in business relations as well. Let us take a deeper dive into the literature tackling this issue: research developed in business (i.e., not in laboratories as described above) settings.

### 1.2. Research on mimicry run in business settings

There is a substantial body of literature on mimicry run in business settings. Below we present several examples of such studies.

#### 1.2.1. Temporal aspects of mimicry

In a recent study, the temporal aspects of mimicry and outcomes stemming from it were researched (Kulesza et al., 2022b). In this study, customers of a cable TV company were mimicked (or not) by a technician during installation of cable hardware. The interaction, during which mimicry took place or not, with the confederate lasted for five, ten or fifteen minutes. Service quality was then measured. The experiment showed that mimicry is not only beneficial (replicating laboratory studies), but even a short period (5 min) of mimicry boosts quality judgments showing the impact of mimicry on service quality evaluations. Kulesza and colleagues (Kulesza et al., 2019) extended this line of research to a restaurant setting, in which a waitress mimicked customers. Finally, in the control condition, no mimicry took place. It turned out that mimicking twice increased the average tip than in the control condition. Interestingly, and in line with previously described work, mimicking a patron of a restaurant even once makes the customer more willing to tip the waitress. This effect takes place regardless of whether the waitress mimics the customer at the beginning and/or at the end of the visit, even after a few minutes. Taken together, mimicry shows great potential for boosting the evaluation of service provided by the mimicker toward the mimickee. It is exactly this potential that is the focus of this paper.

#### 1.2.2. Buying tendencies

Finally, mimicry shows great potential to influence buying tendencies (Christie and Chen, 2018). This experiment lasted for over 9 months and tested if patrons of a restaurant mimic orders placed by the person ahead of them, which is exactly what was discovered. Also, customers of a beauty shop tended to be influenced by mimicry (Kulesza et al., 2014b). As dependent variables the amount of money spent in the store, as well as satisfaction with the service and willingness to return to the store in the future was measured. It turned out that mimicry made participants more eager to spend money on shopping (101.34 PLN) than in the control condition (41 PLN). Sales also varied depending on the attractiveness of the saleswoman.

In an experiment run in a store selling electronic devices orders were mimicked (Jacob et al., 2011). For example, if the patron said to the clerk “Can you help me buy an MP3 player?,” the salesperson would reply: “Of course I can help you buy an MP3 player.” In the control condition, a salesperson would provide only a short answer (e.g., “of course”). As in the beauty shop, it was shown that mimicked customers were more likely to spend more on products, especially those recommended by the mimicker. Moreover, despite buying products, both the salesperson and store received better evaluations from customers/mimickees. These results suggest that the effects of mimicry are not only direct, i.e., restricted only to the mimicker, but also may have an indirect effect, spilling over to the entire company/organization that the mimicker represents.

### 1.3. The present studies

Mimicry in both laboratory and natural settings creates trust (Maddux et al., 2008; Swaab et al., 2011) and rapport (Muir et al., 2020), it also influences purchasing decisions (Tanner et al., 2008; Jacob et al., 2011; Stel et al., 2011a; Kulesza et al., 2014b, 2017, 2019, 2022b; Christie and Chen, 2018) by eliciting susceptibility to the persuasiveness of the mimicker (Van Swol, 2003; Drury and Van Swol, 2005). On this grounds, it may be assumed that research on mimicry and its spillover potential has focused mainly on products, and only partially considered the crucial aspect of spreading the benefits of mimicry to the entire company/organization. Among the first investigations on this issue, Jacob et al. (2011) discovered that a mimicker, despite selling more products to the mimickee, can create a positive evaluation of the entire company/organization that the mimicker represents. Thus, based on Jacob et al. (2011) work, we present a conceptual replication of this result.

We carried out a Pretest, and a Main Experiment where we tested whether the benefits of verbal mimicry may not only affect the partners in the dyad (client - clerk dyad), but may also spillover to other aspects, not related to the interaction where the mimicry was present (opinion and willingness to return to the organization/company; vs. previously: directly on products introduced by the mimicker/clerk). Without a doubt, empirical verification of the above issue is vital for the entire hospitality management industry. A simple mimicry mechanism can generate greater profits not only for the clerk/employee/mimicker, but it can propagate to the entire organization/company in general, which may generate higher profits.

Databases are available at the Open Science Framework (OSF; https://osf.io/kdj4z/). All studies were approved by the local ethics committee (07/P/05/2021).

## 2. Pretest

### 2.1. Method

#### 2.1.1. Participants

46 clients (31 women, 15 men, 0 non-binary persons) of a large popular store (age ranging from 18 to 80; *M* = 44.69, *SD* = 15.96) took part in a Pretest. We did not exclude any participant from the analyses or any data. Using a sensitivity power analysis in G*Power 3.1.9.6 (Faul et al., 2007), we found that with 46 participants, the smallest effect size we could detect at 80% power (α = 0.05) would be Cohen’s *d* = 0.84. There was no missing data.

A preliminary statistical analysis demonstrated that the gender distribution was equal (χ^2^(1, *N* = 46) = 10.9, *p* = 0.001). Participants were randomly assigned to one of two between-subject conditions: no-mimicry (*n* = 23; females = 16, males = 7), and mimicry (*n* = 23; females = 15, males = 8).

#### 2.1.2. Procedure and materials

The Pretest was conducted at a customer service point in one of the biggest, international and most popular franchised grocery stores in the country of research, which offers mainly food, but electronics to some extent as well. Clients who became participants approached the office to complain about, for example, defective products.

During the conversation about the complaint, clients were randomly assigned to two conditions. Following Kulesza et al.’ (2014a) guidelines, in the first condition (no mimicry), answers of the female employee, who was blind to the hypotheses, represented a full understanding of the client’s statements: “yes,” “clear,” and “done.” In the second condition (mimicry), the clerk/confederate employed verbal mimicry by repeating the syntax and words used by the client. If a client said, for example, “The TV I bought in this store stopped working after a week of use,” the customer officer replied: “The point is that the TV you bought from our store stopped working after a week of use.” When the conversation about the complaint ended, the clients were asked to fill in a short questionnaire.

To check whether the spillover effect may have taken place, participants were asked to rate their agreement on 7-point rating scales (1 = *definitively negative*; 7 = *definitely positive*). The questionnaire was based on two sub-scales: two items expressing direct effects of mimicry; employee kindness (“*How do you evaluate the kindness of the employee dealing with your complaint?*”) and employee evaluation (“*How do you evaluate the work of the person dealing with your complaint?*”) as well as two items expressing indirect effects of mimicry; opinion about the store (“*What is your opinion about this store?*”) and willingness to return to the store (“*How do you assess your willingness to return to this store?*”).

In order to obtain an indicator of direct effects of mimicry, we computed the mean of two questions considering *Employee kindness* and *Employee evaluation.* The reliability for this scale was *r_Spearman-Brown_* = 0.61.

In contrast, in the case of indirect effects of mimicry we computed the mean of two questions considering *Opinion about the store* and *Willingness to return to the store.* The reliability for this scale was *r_Spearman-Brown_* = 0.60.

### 2.2. Results

The normality of the distribution of the analyzed parameters was assessed using the Shapiro–Wilk test. Normality tests along with descriptive statistics were calculated and reported for each group separately (see Table 1). Additionally, correlations between analyzed parameters were also calculated. All correlations were positive and ranged from *rho* = 0.210 to *rho* = 0.873. Spearman’s *rho* correlation matrix, along with significance levels and confidence intervals, can be found in Table 2.

**Table 1:** Descriptive statistics of all study variables within the no mimicry and mimicry group (Pretest).

Variables	Descriptive statistics										
									Shapiro–Wilk		
	*M*	*SD*	*Me*	*Mo*	*Sk.*	*Kurt.*	*Min.*	*Max.*	*W*	*df*	*p*
No mimicry											
Store employee kindness	5.61	1.50	6.00	7.00	−0.58	−1.13	3.00	7.00	0.821	23	<0.001
Store employee evaluation	5.61	1.08	5.00	5.00	0.17	−1.33	4.00	7.00	0.830	23	0.001
Indicator of direct effects of mimicry	5.61	1.04	6.00	6.00	−0.61	−0.33	3.50	7.00	0.927	23	0.095
Opinion about the store	5.09	1.59	5.00	7.00	−0.01	−1.62	3.00	7.00	0.849	23	0.003
Willingness to return to the store	5.57	1.31	5.00	5.00	−0.42	−0.74	3.00	7.00	0.862	23	0.005
Indicator of indirect effects of mimicry	5.33	1.19	5.50	4.50	−0.23	−0.52	3.00	7.00	0.937	23	0.155
Mimicry											
Store employee kindness	6.48	0.79	7.00	7.00	−1.13	−0.33	5.00	7.00	0.661	23	<0.001
Store employee evaluation	6.35	0.65	6.00	6.00	−0.48	−0.54	5.00	7.00	0.769	23	<0.001
Indicator of direct effects of mimicry	6.41	0.63	6.50	6.50	−1.11	0.39	5.00	7.00	0.810	23	<0.001
Opinion about the store	6.04	1.15	6.00	7.00	−0.83	−0.65	4.00	7.00	0.768	23	<0.001
Willingness to return to the store	6.35	0.78	7.00	7.00	−0.72	−0.89	5.00	7.00	0.752	23	<0.001
Indicator of indirect effects of mimicry	6.20	0.81	6.50	7.00	−0.92	−0.12	4.50	7.00	0.852	23	0.003

**Table 2:** Spearman’s *rho* correlation matrix between all individual factors with confidence intervals (Pretest).

Variable	1	2	3	4	5
1. Store employee kindness	–	–	–	–	–
2. Store employee evaluation	0.378**	–	–	–	–
	[0.06, 0.66]				
3. Indicator of direct effects of mimicry	0.841***	0.780***	–	–	–
	[0.72, 0.91]	[0.58, 0.92]			
4. Opinion about the store	0.400**	0.315*	0.418**	–	–
	[0.08, 0.67]	[0.00, 0.59]	[0.13, 0.65]		
5. Willingness to return to the store	0.21	0.348*	0.276	0.401**	–
	[−0.09, 0.51]	[0.03, 0.62]	[−0.05, 0.56]	[0.11, 0.67]	
6. Indicator of indirect effects of mimicry	0.345*	0.367*	0.402**	0.873***	0.781***
	[0.03, 0.63]	[0.06, 0.63]	[0.1, 0.66]	[0.76, 0.94]	[0.65, 0.88]

Values in square brackets indicate the 95% confidence interval for each correlation. * indicates *p* < 0.05. ** indicates *p* < 0.01. *** indicates *p* < 0.001.

Then, a series of four *U* Mann–Whitney tests (for each question separately) was run to check the efficiency of the verbal mimicry manipulation. The mimicry manipulation had a positive impact on every considered variable (question). All comparisons turned out to be statistically significant (all *p*s < 0.045). The pattern of these results is presented in Supplementary Material 1.

Next, a series of four (for each question separately) between-subject; experimental condition (2: mimicry vs. no mimicry) x participant gender (2: female vs. male) ANOVAs was run. This analysis indicated that participant gender did not differentiate the results in any way. The pattern of these results is presented in Supplementary Material 2.

#### 2.2.1. Direct and indirect effects of mimicry

In order to check the efficiency of the verbal mimicry manipulation within the indicator of direct and indirect effects of mimicry, a mixed model consisting of one between-subject factor—experimental condition (2: mimicry vs. no mimicry)—and one within-subject factor— directness (2: direct effects of mimicry vs. indirect effects of mimicry) was run.

This analysis revealed an insignificant main effect of directness, *F*(1, 44) = 2.92, *p* = 0.095.

The main effect of the experimental condition was significant, *F*(1, 44) = 12.48, *p* = <0.001, η_p_^2^ = 0.22, 90% CI [0.06, 0.38] indicating that the average participant’s evaluation was overall higher in the mimicry condition (*M* = 6.30, *SD* = 1.14) than in the no mimicry condition (*M* = 5.47, *SD* = 1.14); *t*(44) = 3.53, Cohen’s *d* = 0.89, 95% CI [0.35, 1.43].

The interaction effect of the experimental condition and directness was also insignificant, *F*(1,44) = 0.05, *p* = 0.825. However, in order to test verbal mimicry manipulation efficiency, we decided to perform an exploratory simple main effect analysis with a Bonferroni correction.

This analysis revealed that differences were observed when considering the direct effects. Participants gave higher direct evaluations in the mimicry condition (*M* = 6.41, *SD* = 0.63) than participants in the no mimicry condition (*M* = 5.61, *SD* = 1.04; *t*(44) = 2.89, *p* = 0.031; Cohen’s *d* = 0.85, 95% CI [0.02, 1.69].

The same pattern was also observed when considering the indirect effects. Participants gave higher indirect evaluations in the mimicry condition (*M* = 6.20, *SD* = 0.81) than participants in the no mimicry condition (*M* = 5.33, *SD* = 1.19; *t*(44) = 3.12, *p* = 0.015; Cohen’s *d* = 0.92, 95% CI [0.08, 1.76]).

There were no significant differences in the mimicry condition. Mimicked participants indicated their evaluations at the same level when considering the direct and indirect effects (*p* = 0.999).

There were also no significant differences in the control condition. Not mimicked participants indicated their evaluations at the same level when considering the direct and indirect effects (*p* = 0.999).

**Figure 1:**
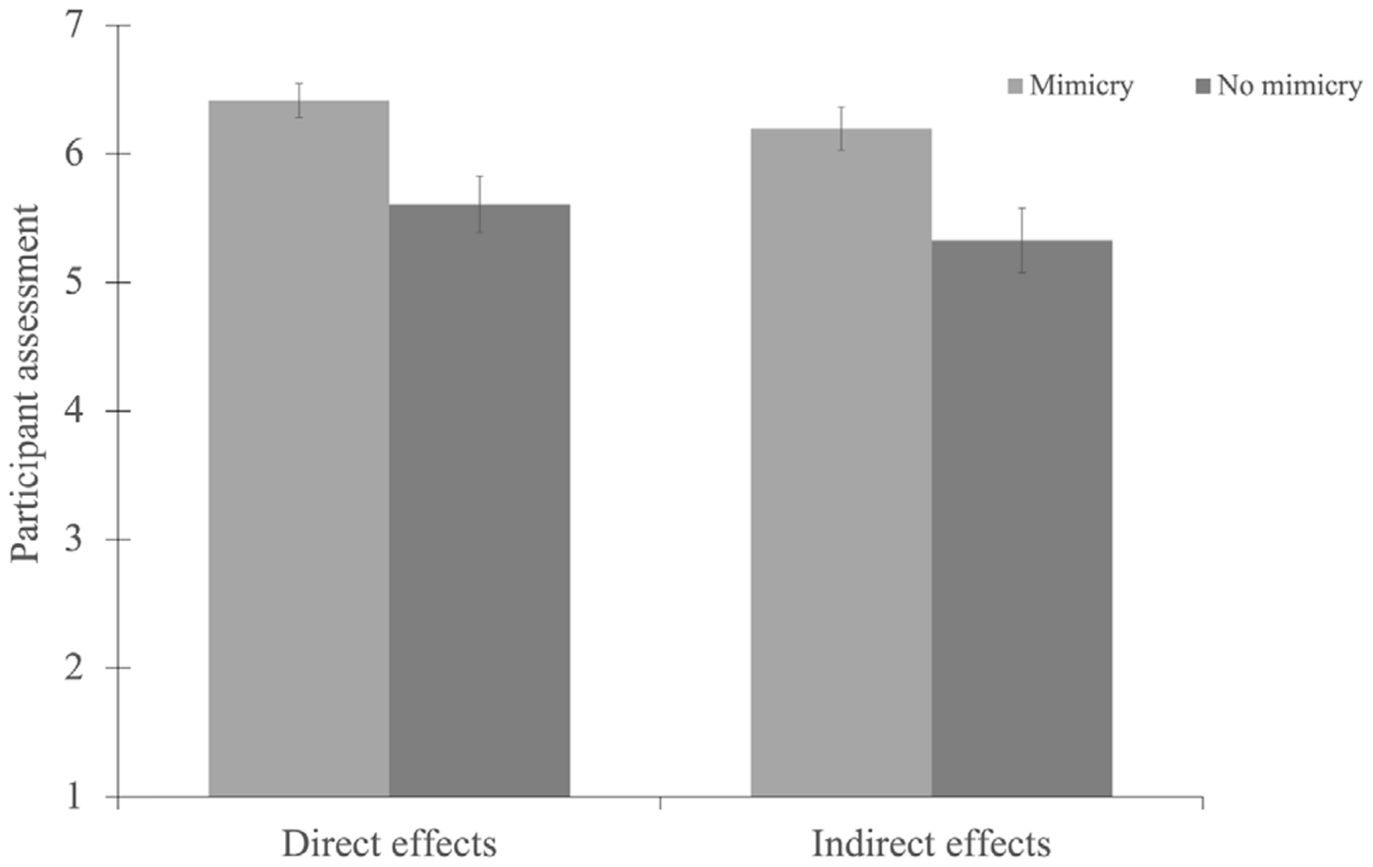
Participants’ evaluation in the Pretest as a function of experimental condition and directness. Bars represent mean values. Error bars represent standard errors of mean.

The pattern of results is visualized in Figure 1.

### 2.3. Discussion

The results of the Pretest showed that people who were subjected to verbal mimicry assessed the mimicker’s kindness more positively, and also made a more positive evaluation of the employee’s work. What is more, this effect was in no way dependent on the gender of the participant. Of particular interest, it was observed that the influence of mimicry reached not only the employee/mimicker, but there was also a spillover of the mimicry to the employee’s organization/company.

In line with research (Kulesza et al., 2017) in which mimicry led to a more positive evaluation of the taste of unpalatable drinks, in this experiment, this mechanism led to a positive evaluation in the context of submitting a complaint, which usually generates negative emotions and attitudes in clients (Westbrook, 1987; Giese and Cote, 2000; Mattsson et al., 2004; White and Yu, 2005; Choraria, 2013). Such a result implies an essential practical thesis: verbal mimicry is an effective tool that influences not only the employee who uses it, but also resonates with a positive overall service outcome even during emotionally unpleasant circumstances.

However, even if these results seem to be promising in a business context, the Pretest is not free from caveats. In order to talk about the reliability of the effect, it is necessary to eliminate the obvious methodological shortcomings in a subsequent study. First, the sample size was far from satisfactory. There was two reasons for that relatively small sample size. First, all participants had to approach the office with the same request (complaint; and not many complaints took place; some clients came to collect an invoice, some to change their order, or for a refund, even though the product worked, but the client was not satisfied with it), and second they had to be alone since the presence of other closely related people changes the pattern of results (see Hanks et al., 2016). Nevertheless, in the Main Experiment, this aspect was addressed by recruiting a sufficient number of participants, in order to detect even the smallest effect size observed in the Pretest (Cohen’s *d* = 0.69, see Supplementary Material 2).

Another issue is external validity. The Pretest took place in a situation where participants were making complaints. It is possible that the observed result of the spillover of mimicry resulted from the positive consideration of the complaints. Participants/clients whose complaint was positively assessed not only positively assessed the person examining the complaint (i.e., the mimicker), but also created a more positive opinion about the organization/company in general. In this situation it is impossible to distinguish the positive impact of mimicry on dependent measures from the positive impact of dealing with the complaint (making people happier). Therefore, the Main Experiment was run in a more neutral context: at a hotel reception, where hotel guests were asked to fill in a questionnaire right after they checked in to the hotel.

## 3. Main experiment

### 3.1. Method

#### 3.1.1. Participants

We aimed to recruit a sufficient sample to detect an effect size of Cohen’s *d* = 0.69, with a power of 1−β = 0.95 and an alpha probability of *α* = 0.05. 112 participants are needed to detect such an effect; *a priori* power analysis using G*Power 3.1.9.6 (Faul et al., 2007). To compensate for possible dropouts, we recruited 120 participants/guests of the hotel (56 women, 64 men, 0 non-binary person), with an age ranging from 18 to 76 (*M* = 41.87, *SD* = 13.34). We did not exclude any participant from the analyses or any data. Using a sensitivity power analysis in G*Power 3.1.9.6 (Faul et al., 2007), we found that with 120 participants, the smallest effect size we could detect at 80% power (*α* = 0.05) would be Cohen’s *d* = 0.52. There was no missing data.

A preliminary statistical analysis demonstrated that the gender distribution was equal (χ^2^(1, *N* = 46) = 1.21, *p* = 0.272). Participants were randomly assigned to one of two between-subject conditions: no-mimicry (*n* = 60; females = 25, males = 35), and mimicry (*n* = 60; females = 31, males = 29).

#### 3.1.2. Procedure and materials

The experiment was conducted in a popular three-star franchise hotel in one of the biggest cities in the country. Participants were recruited from guests checking-in to the hotel. During check-in, the receptionist, who was blind to the hypotheses, mimicked (or not) the hotel guests and later asked them about their preferences for the room, board, and form of payment.

During the conversation, guests were randomly assigned to two conditions. As in the Pretest, in the first condition (no mimicry), the receptionist’s answers were limited to signs of full understanding: “yes,” “clear,” and “done.” In the second condition (mimicry), the hotel receptionist employed verbal mimicry by repeating the words and syntax of the guest’s speech. For example, if the guest said, “my reservation for today is a double room” the receptionist would reply, “your reservation for today is a double room.”

After checking-in, the participant/guest was asked to fill in a short questionnaire. As in Pretest, participants were asked to rate their agreement on 7-point rating scales (1 = *definitively negative*; 7 = *definitely positive*). The questions were almost the same as in the Pretest, but minor changes were necessary for the context of the hotel (not the grocery shop). The questionnaire was based on two sub-scales: two items expressing direct effects of mimicry; employee kindness (“*How do you evaluate the kindness of the employee making your check-in at the hotel?*”) and employee evaluation *(“How do you evaluate the work of the employee making your check-in at the hotel?*”) and two items expressing indirect effects of mimicry; opinion about the hotel *(*“*What is your opinion about this hotel?*”) and willingness to return to the hotel *(*“*How do you assess your willingness to return to this hotel?*”).

As in the Pretest, in order to obtain an indicator of direct effects of mimicry we computed the mean of two questions considering *Employee kindness* and *Employee evaluation.* The reliability for this scale was *r_Spearman-Brown_* = 0.79.

In contrast, in the case of indirect effects of mimicry we computed the mean of two questions considering *Opinion about the hotel* and *Willingness to return to the hotel.* The reliability for this scale was *r_Spearman-Brown_* = 0.86.

### 3.2. Results

As in the Pretest, the normality of the distribution of the analyzed parameters was assessed using the Shapiro–Wilk test. Normality tests along with descriptive statistics were calculated and reported for each group separately (see Table 3). Additionally, correlations between analyzed parameters were also calculated. All correlations were positive, significant at *p* < 0.001, and ranged from *rho* = 0.301 to *rho* = 0.927. Spearman’s *rho* correlation matrix, along with significance levels and confidence intervals, can be found in Table 4.

**Table 3:** Descriptive statistics of all study variables within the no mimicry and mimicry group (Main Experiment).

Variables	Descriptive statistics										
									Shapiro–Wilk		
	*M*	*SD*	*Me*	*Mo*	*Sk.*	*Kurt.*	*Min.*	*Max.*	*W*	*df*	*p*
No mimicry											
Hotel employee kindness	3.90	0.97	4.00	4.00	−0.14	−0.31	2.00	6.00	0.904	60	<0.001
Hotel employee evaluation	4.17	0.81	4.00	4.00	0.29	−0.32	3.00	6.00	0.857	60	<0.001
Indicator of direct effects of mimicry	4.03	0.62	4.00	4.00	−0.29	−0.07	2.25	5.00	0.921	60	<0.001
Opinion about the hotel	3.08	1.11	3.00	4.00	0.06	−0.38	1.00	6.00	0.915	60	<0.001
Willingness to return to the hotel	3.07	1.12	3.00	3.00	0.09	−0.15	1.00	6.00	0.928	60	0.002
Indicator of indirect effects of mimicry	3.08	0.97	3.00	3.00	0.31	0.18	1.00	6.00	0.962	60	0.057
Mimicry											
Hotel employee kindness	5.80	0.90	6.00	6.00	−0.17	−0.83	3.00	7.00	0.867	60	<0.001
Hotel employee evaluation	6.18	0.79	6.00	7.00	−0.34	−1.32	5.00	7.00	0.787	60	<0.001
Indicator of direct effects of mimicry	5.99	0.72	6.00	5.50	0.10	−1.33	5.00	7.00	0.883	60	<0.001
Opinion about the hotel	4.57	1.61	4.50	3.00	0.28	−1.37	2.00	7.00	0.863	60	<0.001
Willingness to return to the hotel	4.43	1.92	4.50	7.00	−0.06	−1.3	1.00	7.00	0.905	60	<0.001
Indicator of indirect effects of mimicry	4.50	1.65	4.25	2.50	0.17	−1.40	2.00	7.00	0.904	60	<0.001

**Table 4:** Spearman’s *rho* correlation matrix between all individual factors with confidence intervals (Main Experiment).

Variable	1	2	3	4	5
1. Hotel employee kindness	–	–	–	–	–
2. Hotel employee evaluation	0.661***	–	–	–	–
	[0.54, 0.77]				
3. Indicator of direct effects of mimicry	0.912***	0.903***	–	–	–
	[0.87, 0.94]	[0.86, 0.94]			
4. Opinion about the hotel	0.392***	0.428***	0.457***	–	–
	[0.22, 0.54]	[0.26, 0.57]	[0.29, 60]		
5. Willingness to return to the hotel	0.301***	0.432***	0.397***	0.688***	–
	[0.11, 0.47]	[0.26, 0.59]	[0.21, 0.56]	[0.56, 0.80]	
6. Indicator of indirect effects of mimicry	0.342***	0.445***	0.433***	0.897***	0.927***
	[0.17, 0.50]	[0.28, 0.59]	[0.28, 0.58]	[0.84, 0.94]	[0.89, 0.95]

Values in square brackets indicate the 95% confidence interval for each correlation. *** indicates *p* < 0.001.

Then, a series of four *U* Mann–Whitney tests (for each question separately) was run to check the efficiency of the verbal mimicry manipulation. The mimicry manipulation had a positive impact on every considered variable (question). All comparisons turned out to be statistically significant (all *ps* < 0.001). The pattern of these results is presented in Supplementary Material 3.

Next, a series of four (for each question separate) between-subject; experimental condition (2: mimicry vs. no mimicry) x participant gender (2: female vs. male) ANOVAs was run. This analysis revealed that gender effects were either insignificant or indicated a relatively small effect size. The exception was willingness to return to the store. In this case, the mimicry effect was particularly strong among men (*p* < 0.001; Cohen’s *d* = 0.87, 95% CI [0.46, 1.24]). The pattern of these results is presented in Supplementary Material 4.

#### 3.2.1. Direct and indirect effects of mimicry

As in the Pretest, in order to check the efficiency of the verbal mimicry manipulation within the indicator of direct and indirect effects of mimicry, a mixed model consisting of one between-subject factor—experimental condition (2: mimicry v., no mimicry)—and one within-subject factor— directness (2: direct effects of mimicry vs. indirect effects of mimicry) was run.

This analysis revealed a significant main effect of directness, *F*(1, 118) = 102.95, *p* < 0.001, η_p_^2^ = 0.47, 90% CI [0.37, 0.55] indicating that participants gave overall higher direct evaluations (*M* = 5.01, *SD* = 1.19) than indirect evaluations (*M* = 3.79, *SD* = 1.53; *t*(118) = 10.15, Cohen’s *d* = 1.14, 95% CI [0.88, 1.41].

The main effect of the experimental condition was also significant, *F*(1, 118) = 121.04, *p* = <0.001, η_p_^2^ = 0.51, 90% CI [0.4, 0.58] indicating that the average participant’s evaluation was overall higher in the mimicry condition (*M* = 5.25, *SD* = 1.1) than in the no mimicry condition (*M* = 3.55, *SD* = 1.10; *t*(118) = 11.01, Cohen’s *d* = 1.58, 95% CI [1.23, 1.93].

The interaction effect of the experimental condition and directness was significant, *F*(1, 118) = 4.88, *p* = 0.029, η_p_^2^ = 0.04, 90% CI [0.01, 0.11]; thus, we performed a simple main effect analysis with a Bonferroni correction.

This analysis revealed that differences were observed when considering the direct effects. Participants gave higher direct evaluations in the mimicry condition (*M* = 5.99, *SD* = 0.72) than participants in the no mimicry condition (*M* = 4.03, *SD* = 0.62; *t*(118) = 10.02, *p* < 0.001; Cohen’s *d* = 1.83, 95% CI [1.25, 2.41]).

The same pattern was also observed when considering the indirect effects. Participants gave higher indirect evaluations in the mimicry condition (*M* = 4.5, *SD* = 1.65) than participants in the no mimicry condition (*M* = 3.08, *SD* = 0.97; *t*(118) = 7.29, *p* < 0.001; Cohen’s *d* = 1.33, 95% CI [0.79, 1.87]).

There were also significant differences in the mimicry condition. Verbally mimicked participants indicated their evaluations as higher when considering direct effects, than when considering indirect effects; *t*(118) = 8.74, *p* < 0.001; Cohen’s *d* = 1.39, 95% CI [0.91, 1.88]).

Statistically significant differences were also observed in the control condition. Not mimicked participants again indicated their evaluations as higher when considering direct effects, than when considering indirect effects; *t*(118) = 5.61, *p* < 0.001; Cohen’s *d* = 0.9, 95% CI [0.44, 1.35].

**Figure 2:**
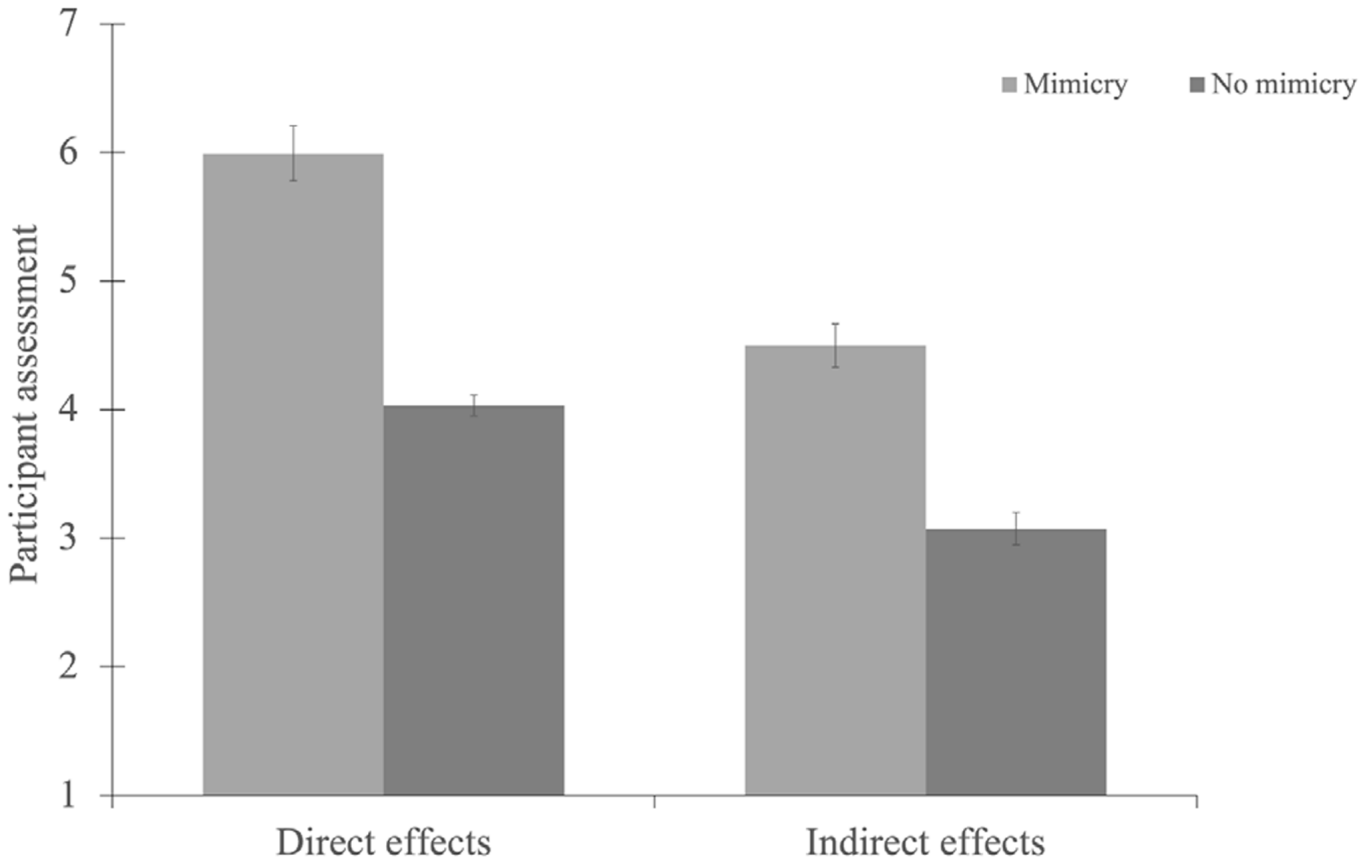
Participants evaluation in the Main Experiment as a function of experimental condition and directness. Bars represent mean values. Error bars represent standard errors of mean.

The pattern of results is visualized in Figure 2.

### 3.3. Discussion

Again, and consistent with the Pretest, participants who were subjected to verbal mimicry assessed the mimicker’s kindness more positively, and also made a more positive evaluation of the employee’s work. Furthermore, the influence of mimicry reached not only the mimicker, but (as in the Pretest) it propagated to the employee’s organization/company. This effect appears to be independent of gender. All gender effects appeared to be either insignificant or indicated a relatively small effect size. The one exception was the willingness to return to the hotel (see Supplementary Material 4). In this case, the mimicry effect was particularly strong for males. However, we want to clarify that gender differences were not the main focus of our study. Moreover, this result in no way affects our conclusions but is nevertheless very interesting. However, with the present data in hand, we can only speculate why this effect appeared. One explanation may be that experiments were conducted in different environments. The Pretest was conducted in a grocery store in a negative context of complaints. The Main Experiment in a hotel was conducted in at least a neutral or even positive context, since the participant self-selected the hotel. The second explanation may be due to different experimenters conducting the Pretest and Main Experiment. Finally, the mixed model revealed that the indirect effects of mimicry are overall weaker than the direct effects. Additionally, this experiment addressed the issues of inadequate sample size, with increased external validity.

## 4. General discussion

The literature demonstrates that mimicry positively influences the perception of the mimicker (e.g., Chartrand and Bargh, 1999; Kulesza et al., 2022a), and this effect can spillover to the products (Tanner et al., 2008; Kulesza et al., 2017), and to the entire company/organization that the mimicker represents (Jacob et al., 2011). The presented studies correspond to the research gap related to mimicry and its spillover potential by systematically verifying, in different contexts, the issue of spillover effects of mimicry and conceptually replicating the result previously reported by Jacob et al. (2011).

In two consecutive studies it was shown that people who were verbally mimicked were more satisfied with the service (which is in line with previous research), even in the negative context of dissatisfaction like complaining about defective products: this effect also spilled over to the organization/company that the mimicker represented.

From a more general perspective, the results support previous findings (e.g., Tanner et al., 2008; Jacob et al., 2011; Kulesza et al., 2017) which show that spillover of mimicry is an important phenomenon from the perspective of marketing/business psychology. Previously it was shown that mimicry, despite aiding in selling more products to the mimickee, can also create a positive evaluation of the entire company/organization that mimicker represents (Jacob et al., 2011). Moreover it was also shown that the spillover effect was present when consumers’ opinions were measured concerning an isotonic drink. Participants were not aware that the person who ran the marketing study mimicked their words and gestures. As a consequence of mimicry manipulation, participants changed their evaluation of the drink: it became much more positive (Tanner et al., 2008). In the context of drinks, it was also shown that poor-tasting beverages can, in terms of mimicry manipulation, become tasty drinks that are worth buying (Kulesza et al., 2017).

### 4.1. Why does it work and how? Social glue as a meta consequence of mimicry

Thus, keeping in mind the works cited above, we present a new quality to this phenomenon: the effects of mimicry can spillover to the entire organization/company (not only to the products) that the mimicker represents, not merely in neutral, but also in negative contexts.

On this grounds, the general question emerges: why does it work and how? Mimicry spills over across the species and it is almost unimaginable to think of an animal in which mimicry is not inherited. Animals mimic each other as a basic survival mechanism. Birds mimic the movements (Voelkl et al., 2015) of, for example, the leader, so the flock consumes less energy when flying large distances, and fish schools are not free from mimicry behavior either (Pitcher, 1979; Partridge et al., 1980; Parrish et al., 2002). Humans are also deeply embedded and benefit from this behavior from the first hours after birth (Meltzoff and Moore, 1983). We spontaneously and intentionally mimic others (Arnold and Winkielman, 2020): we mimic speech utterances (Webb, 1969; Giles and Powesland, 1975; Cappella and Planalp, 1981; Levelt and Kelter, 1982), nonverbal cues (Chartrand and Bargh, 1999), and facial expressions of emotions (Hess et al., 2000), which leads to numerous benefits for both parties of the interaction leading to a conclusion that mimicry is a “social glue” (Lakin et al., 2003; Dijksterhuis, 2005). Two main differences may be drawn from the aforementioned animal kingdom examples. First, contrary to other animals, we mimic not to survive but to socially thrive. Second, while mimicry among animals is oftentimes researched in groups, psychology research mostly concentrates on dyads. The present paper unpacks both issues by researching the social glue phenomenon for more than a mimicking dyad, proving the existence of a spillover effect of mimicry.

As mentioned in the Introduction, on the basis of clinical psychology, it was discovered that mimicking mannerisms leads to better understanding and increased emotional rapport between client and therapist, which is of course a pillar for successful therapy (Charny, 1966; Lafrance and Broadbent, 1976; Sherer and Rogers, 1980; Ramseyer and Tschacher, 2011; Paulick et al., 2018). Secondly, on the basis of social psychology it was reported that when learning social norms, behaviors attached to specific figures are learned by imitating the behaviors of others (Bandura, 1962). Furthermore, it was discovered that this phenomenon, named the chameleon effect (Chartrand and Bargh, 1999), leads to benefits for the mimickerincreased liking and more favorable assessment of the interaction with the mimicker.

In a series of experiments by Van Swol (2003), it was shown that mimicry increased the perception of persuasiveness. Further studies have also shown that mimicry elicits trust between both parties of the interaction (Swaab et al., 2011) even to the point of disclosing personal, and sometimes possibly troubling information. On the other hand, we tend to decrease mimicry while interacting with immoral interlocutors (Menegatti et al., 2020). From the perspective of changes in the perception of the social environment it has been shown that the mimickee starts to perceive the social surrounding as a more just and fair place (Stel et al., 2013), and is even more eager to support parties declaring more pro-socially oriented political programs (Stel and Harinck, 2011). Similarly, in Stel et al.’s (2011b) studies, mimicked participants were more socially/group-oriented whilst no-mimicry condition led to self/individualistic perception of self/others. In the same vein, excluded/ostracized participants in future interactions threw themselves into mimicry behavior to regain the feeling of belongingness and connectedness (Lakin and Chartrand, 2013).

In the same vein, mimicry creates in the mimickee a greater tendency to provide help and other prosocial behaviors, which is a strong signal for the claim that mimicry bonds dyads in which mimicry was present (e.g., Van Baaren et al., 2004; Kulesza et al., 2014a). This tendency to present prosocial behaviors as a result of mimicry was reported not only among children (Kirschner and Tomasello, 2010) but also among adults (e.g., Guéguen et al., 2011; Müller et al., 2012).

The review of the body literature on mimicry clearly shows that mimicry leads not only to an increased tendency to present prosocial behaviors but also to other numerous benefits for the mimicker and mimickee. For example, being mimicked can lead to a positive evaluation of the mimicker (Lakin et al., 2003; Dignath et al., 2018). In the same vein, people mimic more when the target is perceived more positively (Likowski et al., 2008; Stel et al., 2010; Blocker and McIntosh, 2016). Additionally, mimicry can be modulated by a wide range of social cues, including group membership (Yabar et al., 2006; Bourgeois and Hess, 2008; Losin et al., 2012), motivation to affiliate (Lakin et al., 2008; Over and Carpenter, 2009), and the mimicking target’s attractiveness (Karremans and Verwijmeren, 2008; Van Leeuwen et al., 2009).

From this meta-perspective it is clear that mimicry is deployed to build affiliation with others (Chartrand and Bargh, 1999; Lakin et al., 2003) and is a good predictor of individuals’ bonding (Fujiwara et al., 2020). Looking at all of these results may lead to no surprise that mimicry was coined “a social glue” that binds dyads (Lakin et al., 2003; Dijksterhuis, 2005).

Thus, answering the question of “why does it work and how?,” which was posed at the beginning of this section, we stipulate that through mimicry we create and maintain bonds with social surroundings, and not only with humans, but the features they represent. Our results join this line of reasoning. Human beings as social animals imitate as well as like to be imitated, and it seems that mimicry is responsible for one of the most important mechanisms responsible for making us “social animals.”

### 4.2. Limitations and future directions

Undoubtedly, these studies are not free from methodological shortcomings. In both studies, for the vast majority of the dependent variables, no normal distribution was achieved. Future research may try to eliminate this by trying three solutions. Firstly, by using a different measurement tool that would generate more variance in the responses. Secondly, by extending the used scale which would include additional items. Thirdly, by increasing the sample size (although the second study addressed this issue, it is still possible that the increase from the Pretest was not satisfactory).

Another issue is using only a declaration measurement. As is already known, declarations often do not predict real behavior (e.g., Grzyb and Dolinski, 2017), thus subsequent studies may use a behavioral dependent variable. In other words, it would be worthwhile for future studies to use a measurement that includes whether the mimickee/client indeed uses the services of the organization/company in the future, as declared.

One should keep in mind that the studies were conducted in an uncontrolled natural setting. As a consequence, there was high variability of the average duration of employee/mimicker - client/mimickee interaction. In the future, priority should be given to the number of questions asked in the interview, as well as the duration of the interaction. This can be achieved by replicating this effect in a more controlled environment, for example, university lab settings, making the picture more complete.

Ultimately, our research is limited only to the verbal context. Subsequent studies should use other forms of mimicry, such as nonverbal, or double mimicry (simultaneous verbal and nonverbal mimicry). Such a concept could determine if the spillover effect is limited only to the verbal level, or whether an additive effect will be observed.

### 4.3. Conclusions and practical implications

This line of studies clearly shows a clear, replicable spillover effect of mimicry. Benefits are granted by the mimickee not only to the mimicker but also to the organization/company she/he represents. This, in turn, can be an effective technique for creating a positive impression of the organization/company/chain, in both neutral and negative circumstances, which underlines the universal application potential of this phenomenon.

With a simple social mechanism, we can improve the perception of employees and their organization/company, which might lead to a higher willingness to use the organization in the future. Therefore, it is in the best interest of the management of organizations/companies to educate and train employees in using verbal mimicry with their clients. This effect, in turn, may generate greater profits for the entire organization/company in general.

## Data availability statement

The datasets presented in this study can be found in online repositories. The names of the repository/repositories and accession number (s) can be found below: Databases are available at the Open Science Framework (OSF; https://osf.io/kdj4z/).

## Ethics statement

The studies involving human participants were reviewed and approved by the ethics committee of the SWPS University of Social Sciences and Humanities in Wroclaw, Poland (07/P/05/2021). Written informed consent for participation was not required for this study in accordance with the national legislation and the institutional requirements.

## Author contributions

WK, DD, and TG: conceptualization and methodology. PM: formal analysis and visualization. WK, DD, and PM: investigation, writing—original draft preparation, and writing—review and editing. WK: resources and funding acquisition. JB and PB: data curation. WK and PM: project administration. All authors contributed to the article and approved the submitted version.

## Funding

This research was supported by NCN (Narodowe Centrum Nauki – Polish National Science Centre), Preludium Bis 1 grant, granted to WK (number: 2019/35/O/HS6/00420). Publication (open access fee) was supported financially by the Psychology Department (Wroclaw) of SWPS University.

## Conflict of interest

The authors declare that the research was conducted in the absence of any commercial or financial relationships that could be construed as a potential conflict of interest.

## Publisher’s note

All claims expressed in this article are solely those of the authors and do not necessarily represent those of their affiliated organizations, or those of the publisher, the editors and the reviewers. Any product that may be evaluated in this article, or claim that may be made by its manufacturer, is not guaranteed or endorsed by the publisher.
