# Supplementary material for: The chameleon effect in customer relationship management: Experiments on the spillover effects of mimicry in natural settings of a chain hotel and a chain grocery shop
Source: Front Psychol. 2023 Mar 14;14:1016125. doi: 10.3389/fpsyg.2023.1016125 (PMC10043486; doi:10.3389/fpsyg.2023.1016125)
Supplement: Supplementary file 1 [file Data_Sheet_1.docx]

***Supplementary Material 1***

**The Chameleon Effect in Customer Relationship Management: Experiments on the Spillover Effects of Mimicry in Natural Settings of a Chain Hotel and a Chain Grocery Shop**

**Wojciech Kulesza, Dariusz Dolinski, Paweł Muniak^*^, Joanna Borkowska, Polina Bibikova, Tomasz Grzyb**

*** Correspondence:** Paweł Muniak, [pmuniak@swps.edu.pl](mailto:pmuniak@swps.edu.pl)

**Supplementary Table.** U Mann-Whitney Test of Each of the Four Variables (Questions) Within the No Mimicry and Mimicry Group (Pretest).

|  | Experimental group | |  |  |  |  |  |  |
| --- | --- | --- | --- | --- | --- | --- | --- | --- |
|  | No mimicry | Mimicry |  |  |  |  |  |  |
| *Variables* | *M_rank_* | *M_rank_* | *Mann-Whitney U* | *Wilcoxon W* | *Z* | *p* | *r*_biserial_ | *r*_biserial_ 95% CI |
| Store employee kindness | 19.87 | 27.13 | 181.00 | 457.00 | -2.01 | .045 | 0.32 | [-0.01; 0.58] |
| Store employee evaluation | 18.89 | 28.11 | 158.50 | 434.50 | -2.45 | .015 | 0.4 | [0.09; 0.64] |
| Opinion about  the store | 19.59 | 27.41 | 174.50 | 450.50 | -2.06 | .041 | 0.34 | [0.02; 0.6] |
| Willingness to  return to the store | 19.59 | 27.41 | 174.50 | 450.50 | -2.1 | .037 | 0.34 | [0.02; 0.6] |
