# Supplementary material for: The chameleon effect in customer relationship management: Experiments on the spillover effects of mimicry in natural settings of a chain hotel and a chain grocery shop
Source: Front Psychol. 2023 Mar 14;14:1016125. doi: 10.3389/fpsyg.2023.1016125 (PMC10043486; doi:10.3389/fpsyg.2023.1016125)
Supplement: Supplementary file 2 [file Data_Sheet_2.docx]

***Supplementary Material 2***

**The Chameleon Effect in Customer Relationship Management: Experiments on the Spillover Effects of Mimicry in Natural Settings
of a Chain Hotel and a Chain Grocery Shop**

**Wojciech Kulesza, Dariusz Dolinski, Paweł Muniak^*^, Joanna Borkowska, Polina Bibikova, Tomasz Grzyb**

*** Correspondence:** Paweł Muniak, [pmuniak@swps.edu.pl](mailto:pmuniak@swps.edu.pl)

A series of four (for each question separately) between-subject; experimental condition (2: mimicry vs. no mimicry) x participant gender (2: female vs. male) ANOVAs (Pretest).

**Employee kindness**

The ANOVA revealed a significant main effect of experimental condition, *F*(1, 42) = 4.06, *p* = .050, η_p_^2^ = .09, 90% CI [0.01, 0.24] indicating that participants assessed employee kindness significantly higher in the verbal mimicry condition (*M* = 6.48, *SD* = 0.7) than in the no mimicry condition (*M* = 5.61, *SD* = 1.5; *t*(44) = 2.46, Cohen's *d* = 0.72, 95% CI [0.12, 1.32]. The main effect of participant gender was not significant, *F*(1, 42) = 0.07, *p* = .797, as well as the interaction effect of the experimental conditions and participant gender, *F*(1, 42) = 0.45, *p* = .506.

**Employee evaluation**

The ANOVA revealed a significant main effect of experimental condition, *F*(1, 42) = 6.47, *p* = .015, η_p_^2^ = .13, 90% CI [0.01, 0.29] indicating that participants rated employee work higher in the verbal mimicry condition (*M* = 6.35, *SD* = 0.65) than in the no mimicry condition (*M* = 5.61, *SD* = 1.08; *t*(44) = 2.82, Cohen's *d* = 0.83, 95% CI [0.23, 1.43]. The main effect of participant gender was not significant, *F*(1, 42) = 0.13, *p* = .724, as well as the interaction effect of the experimental conditions and participant gender, *F*(1, 42) = 0.03, *p* = .867.

**Opinion about the store**

The ANOVA revealed a significant main effect of experimental condition, *F*(1, 42) = 4.71, *p* = .036, η_p_^2^ = .1, 90% CI [0.01, 0.25] indicating that participants rated their opinion about the store higher in the verbal mimicry condition (*M* = 6.04, *SD* = 1.15) than in the no mimicry condition (*M* = 5.09, *SD* = 1.59; *t*(44) = 2.34, Cohen's *d* = 0.69, 95% CI [0.09, 1.28]. The main effect of participant gender was not significant, *F*(1, 42) = 0.07, *p* = .797, as well as the interaction effect of the experimental conditions and participant gender, *F*(1, 42) = 0.45, *p* = .506.

**Willingness to return to the store**

The ANOVA revealed a significant main effect of experimental condition, *F*(1, 42) = 8.51, *p* = .006, η_p_^2^ = .17, 90% CI [0.03, 0.33] indicating that participants assessed their willingness to return to the store higher in the verbal mimicry condition (*M* = 6.35, *SD* = 0.78) than in the no mimicry condition (*M* = 5.56, *SD* = 1.31; *t*(44) = 2.47, Cohen's *d* = 0.73, 95% CI [0.13, 1.32]. The main effect of participant gender was not significant, *F*(1, 42) = 0.07, *p* = .797, as well as the interaction effect of the experimental conditions and participant gender, *F*(1, 42) = 0.45, *p* = .506.
