# Supplementary material for: The chameleon effect in customer relationship management: Experiments on the spillover effects of mimicry in natural settings of a chain hotel and a chain grocery shop
Source: Front Psychol. 2023 Mar 14;14:1016125. doi: 10.3389/fpsyg.2023.1016125 (PMC10043486; doi:10.3389/fpsyg.2023.1016125)
Supplement: Supplementary file 3 [file Data_Sheet_3.docx]

***Supplementary Material 3***

**The Chameleon Effect in Customer Relationship Management: Experiments on the Spillover Effects of Mimicry in Natural Settings of a Chain Hotel and a Chain Grocery Shop**

**Wojciech Kulesza, Dariusz Dolinski, Paweł Muniak^*^, Joanna Borkowska, Polina Bibikova, Tomasz Grzyb**

*** Correspondence:** Paweł Muniak, [pmuniak@swps.edu.pl](mailto:pmuniak@swps.edu.pl)

**Supplementary Table.** U Mann-Whitney Test of Each of the Four Variables (Questions) Within the No Mimicry and Mimicry Group (Main Experiment).

|  | Experimental group | |  |  |  |  |  |  |
| --- | --- | --- | --- | --- | --- | --- | --- | --- |
|  | No mimicry | Mimicry |  |  |  |  |  |  |
| *Variables* | *M_rank_* | *M_rank_* | *Mann-Whitney U* | *Wilcoxon W* | *Z* | *p* | *r*_biserial_ | *r*_b_ 95% CI |
| Hotel employee kindness | 35.62 | 85.38 | 307.00 | 2137.00 | -8.03 | <.001 | 0.83 | [0.75; 0.88] |
| Hotel employee evaluation | 33.59 | 87.41 | 185.50 | 2015.50 | -8.68 | <.001 | 0.89 | [0.85; 0.93] |
| Opinion about  the hotel | 45.76 | 75.24 | 915.50 | 2745.50 | -4.76 | <.001 | 0.49 | [0.32; 0.63] |
| Willingness to  return to the hotel | 48.30 | 72.70 | 1068.00 | 2898.00 | -3.91 | <.001 | 0.41 | [0.22; 0.56] |
