# Supplementary material for: The chameleon effect in customer relationship management: Experiments on the spillover effects of mimicry in natural settings of a chain hotel and a chain grocery shop
Source: Front Psychol. 2023 Mar 14;14:1016125. doi: 10.3389/fpsyg.2023.1016125 (PMC10043486; doi:10.3389/fpsyg.2023.1016125)
Supplement: Supplementary file 4 [file Data_Sheet_4.docx]

***Supplementary Material 4***

**The Chameleon Effect in Customer Relationship Management: Experiments on the Spillover Effects of Mimicry in Natural Settings
of a Chain Hotel and a Chain Grocery Shop**

**Wojciech Kulesza, Dariusz Dolinski, Paweł Muniak^*^, Joanna Borkowska, Polina Bibikova, Tomasz Grzyb**

*** Correspondence:** Paweł Muniak, [pmuniak@swps.edu.pl](mailto:pmuniak@swps.edu.pl)

A series of four (for each question separately) between-subject; experimental condition (2: mimicry vs. no mimicry) x participant gender (2: female vs. male) ANOVAs (Main Experiment).

**Employee kindness**

The ANOVA revealed a significant main effect of experimental condition, *F*(1, 116) = 119.06, *p* < .001, η_p_^2^ = .51, 90% CI [0.4, 0.59] indicating that participants assessed employee kindness significantly higher in the verbal mimicry condition (*M* = 5.8, *SD* = 0.9) than in the no mimicry condition (*M* = 3.9, *SD* = 0.97; *t*(118) = 11.14, Cohen's *d* = 2.03, 95% CI [1.59, 2.47]. The main effect of participant gender was not significant, *F*(1, 116) = 0.3, *p* = .585, as well as the interaction effect of the experimental conditions and participant gender, *F*(1, 116) = 1.55, *p* = .215.

**Employee evaluation**

The ANOVA revealed a significant main effect of experimental condition, *F*(1, 116) = 197.12, *p* < .001, η_p_^2^ = .63, 90% CI [0.54, 0.69] indicating that participants rated employee work higher in the verbal mimicry condition (*M* = 6.18, *SD* = 0.72) than in the no mimicry condition (*M* = 4.17, *SD* = 0.81; *t*(118) = 13.83, Cohen's *d* = 2.52, 95% CI [2.04, 3.0].

The main effect of participant gender was significant too, *F*(1, 116) = 4.21, *p* = .042, η_p_^2^ = .04, 90% CI [0.01, 0.1] indicating that males (*M* = 5.22, *SD* = 1.3) rated employee work higher than females (*M* = 5.12, *SD* = 1.28; *t*(118) = 0.4, Cohen's *d* = 0.07, 95% CI [-0.27, 0.43]. The interaction effect of the experimental conditions and participant gender was not significant, *F*(1, 116) = 0.31, *p* = .578.

**Opinion about the hotel**

The ANOVA revealed a significant main effect of experimental condition, *F*(1, 116) = 35.79, *p* < .001, η_p_^2^ = .24, 90% CI [0.13, 0.34] indicating that participants rated their opinion about the hotel higher in the verbal mimicry condition (*M* = 4.57, *SD* = 1.61) than in the no mimicry condition (*M* = 3.08, *SD* = 1.11; *t*(118) = 5.88, Cohen's *d* = 1.07, 95% CI [0.68, 1.46]. The main effect of participant gender was not significant, *F*(1, 116) = 2.83, *p* = .095, as well as the interaction effect of the experimental conditions and participant gender, *F*(1, 116) = 1.25, *p* = .267.

**Willingness to return to the hotel**

The ANOVA revealed a significant main effect of experimental condition, *F*(1, 116) = 27.3, *p* < .001, η_p_^2^ = .19, 90% CI [0.09, 0.29] indicating that participants assessed their willingness to return to the hotel higher in the verbal mimicry condition (*M* = 4.43, *SD* = 1.92) than in the no mimicry condition (*M* = 3.07, *SD* = 1.12; *t*(118) = 4.77, Cohen's *d* = 0.87, 95% CI [0.46, 1.24]. The main effect of participant gender was significant too, *F*(1, 116) = 11.97, *p* < .001, η_p_^2^ = .09, 90% CI [0.03, 0.18] indicating that males (*M* = 4.13, *SD* = 1.7) assessed their willingness to return to the hotel higher than females (*M* = 3.32, *SD* = 1.63; *t*(118) = 2.64, Cohen's *d* = 0.48, 95% CI [0.12, 0.85].

The interaction effect of the experimental conditions and participant gender was significant, *F*(1, 116) = 4.14, *p* = .044, η_p_^2^ = .03, 90% CI [0.01, 0.1]. A simple main effect analysis with a Bonferroni correction, revealed that differences were observed between males in the verbal mimicry (*M* = 5.21, *SD* = 1.7) and no mimicry condition (*M* = 3.23, *SD* = 1.06; *t*(62) = 5.69, Cohen's *d* = 1.43, 95% CI [0.87, 1.98]). Interestingly, the mimicry effect in this case was not present for females between mimicry conditions (*p* = .186).

Statistically significant differences were also observed in the mimicry condition between males and females (*M* = 3.71, *SD* = 1.85; *t*(58) = 3.26, Cohen's *d* = 0.84, 95% CI [0.32, 1.37]). No statistically significant differences were observed in the no mimicry condition between gender (*p* = .999). Finally, significant differences were observed between males in the mimicry condition and females in the no mimicry condition (*M* = 2.84, *SD* = 1.18; *t*(58) = 5.85, Cohen's *d* = 1.6, 95% CI [0.98, 2.21]). All other comparisons were not significant (all *ps* < .186).
